# Decreased *GATA5* mRNA expression associates with CpG island methylation and shortened recurrence-free survival in clear cell renal cell carcinoma

**DOI:** 10.1186/1471-2407-14-101

**Published:** 2014-02-17

**Authors:** Inga Peters, Natalia Dubrowinskaja, Michael Kogosov, Mahmoud Abbas, Jörg Hennenlotter, Christoph von Klot, Axel S Merseburger, Arnulf Stenzl, Ralph Scherer, Markus A Kuczyk, Jürgen Serth

**Affiliations:** 1 Department of Urology and Urologic Oncology, Hannover Medical School, Carl-Neuberg-Str.1, Hannover 30625, Germany; 2 Department of Pathology, Hannover Medical School, Hannover, Germany; 3 Department of Urology, Eberhard Karls University of Tuebingen, Tuebingen, Germany; 4 Department of Biometry, Hannover Medical School, Hannover, Germany

**Keywords:** *GATA5*, Renal cell carcinoma, mRNA, Prognosis, DNA methylation

## Abstract

**Background:**

GATA-5, a zinc-finger transcription factor and member of the GATA family proteins 1–6, is known to be involved in cellular differentiation. We recently found that tumor-specific hypermethylation of the *GATA5* CpG island (CGI) occurs in renal cell carcinoma (RCC) and is associated with an adverse clinical outcome. In this study, we investigated whether epigenetic *GATA5* alterations may result in changes in *GATA5* mRNA expression levels and correlate with the observed prognostic impact of epigenetic changes in *GATA5* in RCC.

**Methods:**

Quantitative real-time reverse-transcribed polymerase chain reaction was applied to measure relative *GATA5* mRNA expression levels in 135 kidney tissue samples, including 77 clear cell RCC (ccRCC) tissues and 58 paired adjacent normal renal tissue samples. Relative *GATA5* expression levels were determined using the ΔΔCt method and detection of three endogenous control genes then compared to previously measured values of relative methylation.

**Results:**

The mean relative *GATA5* mRNA expression level exhibited an approximately 31-fold reduction in tumor specimens compared with corresponding normal tissues (p < 0.001, paired *t-*test). Decreased *GATA5* mRNA expression was inversely correlated with increased *GATA5* CGI methylation (p < 0.001) and was associated with shortened recurrence-free survival in ccRCC patients (p = 0.023, hazard ratio = 0.25).

**Conclusion:**

*GATA5* mRNA expression is decreased in ccRCC, likely due to gene silencing by methylation of the *GATA5* CGI. Moreover, reduced *GATA5* mRNA levels were associated with a poor clinical outcome, indicating a possible role of *GATA5* for the development of aggressive ccRCC phenotypes.

## Background

Renal cell carcinoma (RCC) is one of the top ten causes of cancer deaths in industrialized countries, and its incidence has consistently increased during the past decades [1]. Clear cell renal cell carcinomas (ccRCCs) are the most frequently occurring histological entity, comprising approximately 75% of all RCC.

As a member of the GATA family of transcription factors, *GATA5* is known to be functionally involved in cellular lineage and cell differentiation during embryonic development of the heart, lung, urogenital tract, and gut epithelium [2]. Altered expression of *GATA5* has been associated with intestinal epithelial cell differentiation [3]. *GATA5* is assumed to be a selective transcriptional regulator of mucin genes in gastrointestinal tissues [4], and regulates the promoter of the sodium-hydrogen exchanger isoform 3 that is expressed in intestinal and renal epithelium via Sp-family transcription factors [5].

Of note, a previous study found *GATA5* hypermethylation and associated epigenetic silencing to be involved in carcinogenesis of gastric and colorectal cancers [6]. Epigenetic alterations of *GATA5* were also described in other tumor tissues and were linked to the development of ovarian, lung, pancreatic, and esophageal cancer [7-10]. In a recent study aimed at identifying new DNA methylation targets in ccRCC, we detected for the first time a tumor-specific hypermethylation of the *GATA5* CpG island (CGI) in RCC [11]. Hypermethylation was also associated with advanced disease and shortened recurrence-free survival (RFS) of patients.

In this study, we asked whether mRNA expression levels of *GATA5* are reduced as suggested by our previous DNA methylation analysis, and if the mRNA levels are associated with adverse clinical parameters, further underlining the relevance of epigenetic *GATA5* alterations in ccRCC carcinogenesis.

## Material and methods

### Tissue specimens

One hundred and thirty-five kidney tissues, including 77 ccRCCs and 58 paired adjacent normal renal tissue samples, were included in this study. RCC tissues were obtained from open or laparoscopic nephrectomies and partial resection. Paired tissue samples with adjacent normal tissue (adN) were obtained from a subgroup of our 77 ccRCCs cohort. Adjacent normal tissues, i.e. morphologically normal kidney were isolated with minimum of 0.5 cm to 2 cm distant from the primary tumor lesion. Samples were snap-frozen in liquid nitrogen immediately following surgery and stored at −80°C. Ethical approval of the university ethical committee (Prof. H. D. Tröger, Hannover Medical School, Carl-Neuberg-Str. 1, Hannover, Germany) and informed consent from all patients were obtained. Localized disease was defined as pT ≤ 2, lymph node involvement and metastasis negative (N0/M0), and grading (G) G1 and 1–2, whereas advanced RCC was defined as pT ≥ 3, N1 and/or M1, and G2-3 and G3. Patients with G2 were assigned to the intermediate risk group and were not considered as a parameter for low vs. high grade group comparisons. Follow up data were available for 35 patients, and RFS was defined as the interval up to the time that disease progression could be detected by computer tomography scans. Clinical and histopathological parameters are summarized in Table 1.

**Table 1:** Clinicopathological parameters of ccRCC patients

**Clinicopathological parameters**		**n**	**%**
Cases in total		77	100
*Sex*	Female	29	38
	Male	48	62
*Median age, years*		64	
*Median tumor size, cm*		5.5	
*Primary tumor classification*	pT1	6	8
	pT1a	19	25
	pT1b	14	18
	pT2	3	4
	pT3	2	3
	pT3a	9	12
	pT3b/c	22	29
	pT4	0	0
	not known	2	3
*Lymph node status*	N0	68	88
	N1	9	12
	N2	0	0
*Status of metastasis*	M0	56	73
	M1	21	27
*Grade*			
- Low risk	G1	14	18
	G1-2	8	10
- Intermediate risk	G2	40	52
- High risk	G2-3	5	6
	G3	10	13
*Localized disease*	pT ≤ 2, N0, M0 and G1; G1-2	34	44
*Advanced disease*	pT ≥ 3 and/or N1, M1 or G2-3; G3	43	56
*Paired samples*		58	75

*Abbreviations*: ccRCC clear cell renal cell carcinoma, n number.

### RNA isolation, cDNA synthesis, and quantitative real-time PCR analysis

Isolation of total RNA from tissue specimens and from renal proximal tubular epithelial cells (RPTEC) as controls, cDNA synthesis, and quantitative real-time PCR analysis (qRT-PCR) were carried out as described previously [12]. Duplicate measurements for qRT-PCR analysis were performed using 384 sample plates, an automated liquid handling system (FasTrans, AnalyticJena, Jena, Germany), and the ABI 7900 Fast Sequence Detection System as described previously [12]. Experimenters were blinded to any patient clinicopathological or survival information. The TaqMan expression assays used were *GATA-5* (Hs00388359_m1), *HPRT1* (Hs99999909_m1), *GUSB* (Hs00939627_m1), and *RPL13A* (Hs03043885_g1) (all assays were from Life Technologies, Foster City, CA, USA). *HPRT1, GUSB,* and *RPL13A* were included as endogenous references. The cDNA obtained from RPTEC primary cell transcripts served as biological controls. For each qRT-PCR run, blank and no-template controls were included. Relative expression levels were calculated using the delta-deltaCT (ΔΔCt) method [13,14], and the SDS 2.3 Manager and dataAssist V2.0 software (Life technologies) as described previously [12]. The endogenous controls, *HPRT1*, *RPL13A* and *GUSB,* were combined by dataAssist V2.0 software and “arithmetic mean” was used as a method of normalization.

### Statistics and survival analysis

Natural logarithms of relative expression (lnRQ) values were used for statistical calculations. All statistics were done using the statistical software, R 2.15.2 [15]. The paired *t-test* was used for statistical analyses of expression differences in paired tumor and adjacent normal tissues samples, whereas univariate Cox regression models were used for statistical analysis of RFS. The threshold for dichotomization of expression values was calculated using the selected rank statistics of R package, which provides the minimum p-value for log rank statistics [15]. P-values < 0.05 were considered to be statistically significant. The Kaplan-Meier method was used for survival analyses.

## Results

### GATA5 mRNA expression is decreased in ccRCC

**Figure 1:**
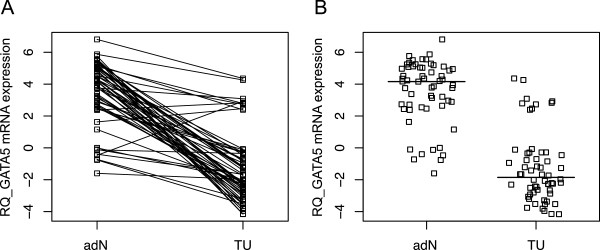
***GATA5 *mRNA expression in paired clear cell renal cell carcinoma and adjacent normal tissues. A)** Comparison of the relative *GATA5* expression (RQ) values in adjacent normal (adN) and tumor (TU) tissues from ccRCC patients (p < 0.001). **B)** Scatterplot analysis illustrating the distribution of relative expression values (RQ) observed for TU and adN in ccRCC specimens. Bold lines indicate the median of relative expression values.

The analyses of relative *GATA5* mRNA expression levels revealed significantly decreased expression in tumor specimens (TU; mean lnRQ = −1.7; ±SD = 1.63) compared with the corresponding adN (mean lnRQ = 1.73; ±SD = 1.32; p < 0.001; paired *t*-test). Figure 1A illustrates the differences in expression values observed for paired tumor and adN, indicating a strong reduction of up to 31-fold in the expression levels, largely in tumor tissues. The comparison of the distribution of relative expression values between both tissue groups showed only a small overlap (Figure 1B).

### Loss of GATA5 mRNA expression correlates with CGI hypermethylation

**Figure 2:**
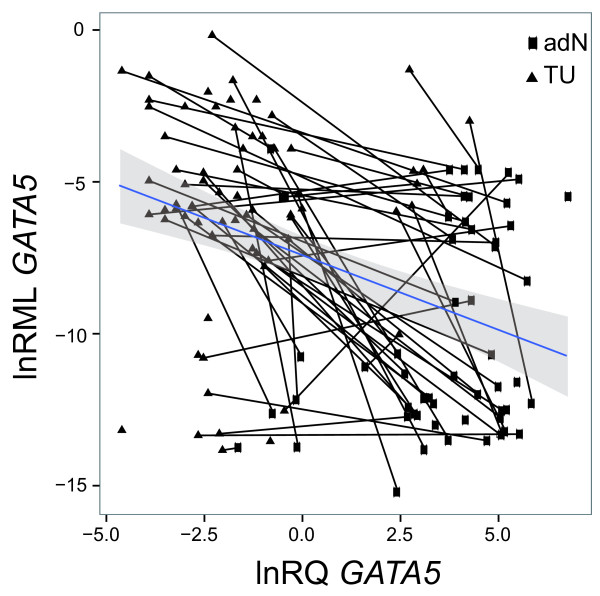
**Association of *GATA5 *CGI methylation and relative mRNA expression in tumor and adjacent normal tissues.** Solid lines connect the subgroup of paired tissues (tumor tissue = solid triangle; adjacent normal = solid squares). The regression line (dashed line) and 95% CI (grey shaded) are presented. Note that tissues exhibiting concurrent high methylation and low mRNA expression can be found in the upper left corner, whereas the occurrence of low methylation and higher relative expression in tissues is displayed in the lower right corner.

We compared lnRQ expression values and natural logarithms relative methylation (lnRML) values of *GATA5* in all samples (Figure 2). Regression analysis revealed an inverse relationship between relative expression levels and methylation of *GATA5* (coefficient of regression = −0.41, p < 0.001). The comparison of paired tissues, indicated by solid lines, shows that high methylation values with concurrent low expression is frequently observed in tumor tissues, whereas corresponding adN samples largely had high expression levels and low methylation.

### GATA5 mRNA expression is associated with tumor diameter

Logistic regression analysis for comparison of tumor subgroups detected a significant difference in mRNA expression levels only for the tumor diameter (p = 0.02, odds ratio = 0.63; 95% CI: 0.42–0.93) whereas other clinicopathological parameters like sex, gender, distant metastasis, lymph node metastasis and tumor grade exhibited no statistically significant association.

### Loss of GATA5 mRNA expression is associated with decreased recurrence-free survival

**Figure 3:**
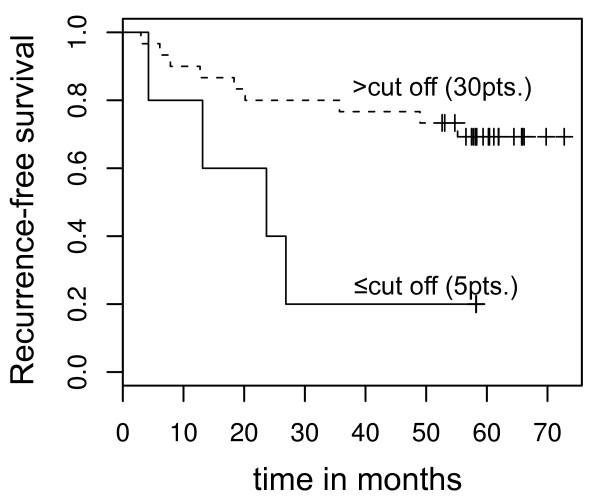
**Kaplan-Meier plot for illustrating recurrence-free survival.** The solid line shows the Kaplan-Meier curve for 5 patients (pts.) with *GATA5* mRNA expression lower than or equal to the cut off of −3.52 (natural logarithm), indicating patients with a shortened recurrence-free survival. The dashed line illustrates the Kaplan-Meier curve for patients with mRNA expression levels above the cut off including 30 pts. Disease progression of ccRCC within a period of approximately two years was observed in seven cases with a low relative mRNA expression level, whereas high *GATA5* mRNA expression phenotypes showed only four progression events within that time interval.

Cox regression survival analyses using a statistically calculated optimum cut off value for relative *GATA5* mRNA expression (lnRQ = −3.52) showed that a lower expression status was associated with increased risk for shorter time to disease recurrence (p = 0.023, hazard ratio (HR) = 0.25, 95% CI: 0.07–0.82; Table 2). Within 30 months, four out of five patients (80%) whose tumor specimens demonstrated expression values below the cut off value were identified with disease recurrence (Figure 3). The status of localized and advanced disease (p = 0.03, HR = 4.18; 95% CI: 1.15–15.2), status of metastasis (p = 0.009, HR = 4.27; 95% CI: 1.43–12.8), and tumor grade (p < 0.001, HR = 9.48; 95% CI: 2.92–30.8) were also shown to be associated with RFS (Table 2). Pairwise bivariate Cox regression analyses were first carried out to investigate whether an association between expression status and clinicopathological parameters and RFS exists. In bivariate statistical models, considering the statuses of advanced disease, metastasis, and tumor grade as covariates, we found in each case that *GATA5* expression was not associated with RFS while mRNA levels were detected as a significant parameter in bivariate Cox regression models including the statuses of lymph node metastasis, age and gender (Table 3). Moreover, we carried out a multivariate analysis demonstrating that mRNA expression of *GATA5* (p = 0.12, HR = 0.32; 95% CI: 0.07–1.34) is not significantly associated with RFS including the covariates status of advance disease (p = 0.29, HR = 0.18; 95% CI: 0.01-4.41), status of metastasis (p = 0.14, HR = 6.60; 95% CI: 0.55–78.68), tumor grade p = 0.002, HR = 0.09; 95% CI: 2.46–56.14, age (p = 0.36, HR = 2.54; 95% CI: 0.05–2.85) and gender (p = 0.39, HR = 2.03; 95% CI: 0.39–10.38).

**Table 2:** **Univariate statistical association of *GATA5 *** **mRNA expression and clinicopathological parameters with recurrence-free survival**

	**p-value°**	**HR**	**95% CI**
*GATA5* mRNA expression	**0.023**	0.25	0.07-0.82
Localized vs. Advanced	**0.030**	4.18	1.15-15.2
Status of metastasis	**0.009**	4.27	1.43-12.8
Tumor grade	**<0.001**	9.48	2.92-30.8
Lymph node status	0.398	1.920	0.42-8.72
Age*	0.155	0.420	0.13-1.38
Gender	0.489	1.510	0.47-0.82

°Univariate Cox regression analysis.

*Dichotomization by the median of parameter.

bold numbers: p-value < 0.05 (statistically significant).

hazard ratio (HR).

confidence interval (CI).

**Table 3:** **Bivariate statistical association of *GATA5 *** **mRNA expression and clinicopathology with recurrence-free survival**

	**p-value°**	**HR**	**95% CI**
*GATA5 mRNA expression*	0.137	0.390	0.11-1.35
Localized vs Advanced	0.080	3.340	0.87-12.9
*GATA5 mRNA expression*	0.113	0.370	0.11-1.27
Status of metastasis	0.036	3.410	1.08-10.8
*GATA5 mRNA expression*	0.511	0.640	0.17-2.41
Tumor grade	0.001	8.320	2.34-29.6
*GATA5 mRNA expression*	**0.032**	0.260	0.08-0.90
Lymph node metastasis	0.657	1.420	0.29-6.77
*GATA5 mRNA expression*	**0.035**	0.270	0.08-0.91
Age*	0.214	0.470	0.14-1.55
*GATA5 mRNA expression*	**0.023**	0.250	0.08-0.83
Gender	0.512	1.480	0.46-4.84

°Bivariate Cox regression analysis.

*Dichotomization by the median of parameter.

bold numbers: p-value < 0.05 (statistically significant).

hazard ratio (HR).

confidence interval (CI).

## Discussion

Members of the GATA1-6 transcription factor family contribute to stem cell differentiation in embryonic tissue, and *GATA5* is involved in intestinal epithelial cell differentiation in adults [3]. Moreover, previous analyses found *GATA5* hypermethylation in human malignancies such as gastric and colorectal cancers and demonstrated that epigenetic silencing of the gene occurred in various human cancer cell lines, providing evidence that *GATA5* alterations may represent epigenetic alterations of wider relevance for carcinogenesis [4,6].

In a recent study aimed at identifying new DNA methylation targets in ccRCC, we detected tumor-specific hypermethylation of the *GATA5* CGI in RCC [11]. Hypermethylation was also associated with advanced disease and shortened RFS of patients, which had not been previously reported for any other human cancer.

Hypermethylation of *GATA5* in RCC indicated that the expression of *GATA5* might be epigenetically silenced in tumor cells, leading to a biologically more aggressive tumor phenotype. In the current study, we demonstrate that *GATA5* mRNA expression is strongly reduced in ccRCC and, moreover, that a subgroup of tissues shows a clear relationship between methylation of the *GATA5* CGI and reduced mRNA expression, indicating that epigenetic silencing of *GATA5* occurs in a substantial fraction of ccRCC. A significant relationship between *GATA5* hypermethylation and reduced *GATA5* mRNA expression within a human tissue, to the best of our knowledge, has not been previously demonstrated. Thus, our results support the notion that epigenetic silencing due to DNA methylation is a relevant process in RCC.

A subgroup of tissues showed only moderate reduction of expression, although *GATA5* methylation was detectable, indicating that other biological mechanisms, e.g. histone alterations, play a role in tumor development in ccRCC. Additional functional investigations are required to clarify these aspects.

*GATA5* methylation is associated with various clinicopathological parameters as well as RFS. Hence, we hypothesized that reduced mRNA expression levels in ccRCC would also show an association with unfavorable clinical parameters. Indeed, we found that decreased *GATA5* mRNA expression is associated with the diameter of tumors and RFS in univariate Cox regression analysis, showing a hazard ratio of 0.25, which resembles the reciprocal hazard ratio observed for the corresponding methylation analysis.

Interestingly, a subset of patients with very low mRNA expression levels also demonstrated a shortened recurrence-free survival in univariate Cox regression analysis. However, taking into account that only a small number of tumors have been identified, these results require future extended evaluation studies including multivariate analyses.

## Conclusion

Decreased *GATA5* mRNA expression in ccRCC may be caused by epigenetic silencing, and is likely associated with a poor clinical outcome. Our results underline the need for further functional studies to characterize the interaction of *GATA5* and cellular signaling in ccRCC with respect to the observed changes in expression and methylation levels, and its association with tumor progression.

## Competing interest

The authors’ declare that they have no competing interest.

## Authors’ contributions

IP wrote the manuscript, prepared the figures and participated in the study design. ND and MK carried out the methylation analyses and participated in the sequence analyses. JH and MK assembled histopathological, clinicopathological and survival data. JH performed isolation and characterization of tissue samples and assembly of patients. MA evaluated the histopathologies of given tissues. MK, CvK, AM and AS assisted with general scientific discussion. JS identified the candidate promoter, conceived of the study, developed the study design and analytical assays, constructed and ran the clinical database, performed statistical analyses together with RS and participated in manuscript preparation. All authors read and approved the final manuscript.

## Pre-publication history

The pre-publication history for this paper can be accessed here:

http://www.biomedcentral.com/1471-2407/14/101/prepub
